# miR-31-3p functions as a tumor suppressor by directly targeting GABBR2 in prostate cancer

**DOI:** 10.3389/fonc.2022.945057

**Published:** 2022-08-18

**Authors:** Sujin Choi, Soonchul Lee, Young-Hoon Han, Junwon Choi, Isaac Kim, Jusung Lee, Hyun-Ju An

**Affiliations:** ^1^ Department of Orthopaedic Surgery, CHA Bundang Medical Center, CHA University School of Medicine, Pangyo-ro, South Korea; ^2^ Division of Radiation Cancer Research, Korea Institute of Radiological and Medical Sciences, Seoul, South Korea; ^3^ Department of Molecular Science and Technology, Ajou University, Yeongtong-gu, South Korea; ^4^ Department of General Surgery, CHA Bundang Medical Center, CHA University School of Medicine, Pangyo-ro, South Korea

**Keywords:** miR-31-3p, GABBR2, prostate cancer, miRNA, tumor suppressor gene

## Abstract

MicroRNAs are key regulators of gene expression in tumorigenesis. In this study, we investigated the tumor-suppressive function of miR-31-3p. Analysis of the Gene Expression Omnibus database revealed that the expression of miR-31-3p in prostate cancer tissues is lower than that in adjacent normal tissues from patients with prostate cancer. Moreover, miR-31-3p induces apoptosis in DU145, PC-3, and LNCap prostate cancer cells, while those transfected with miR-31-3p exhibit significantly decreased cell proliferation, migration, invasiveness, and tumor sphere-forming ability, as determined using the cell counting kit-8, transwell, and sphere-forming assays. Further analysis revealed that GABBR2 is a direct target of miR-31-3p. Within a DU145 xenograft murine model, intratumoral injection of a miR-31-3p mimic suppresses tumor growth. Taken together, the findings of this study suggest that miR-31-3p performs a novel tumor-suppressive function in prostate cancer and may represent a novel target for anti-prostate cancer miRNA therapeutics.

## 1 Introduction

Prostate cancer (PC) is the most common cancer in American men, with an estimated 248,530 new cases and 34,130 deaths in 2021, accounting for 11% of all male cancer-related deaths (1). Although prostate-specific antigen (PSA), has been widely used as a PC biomarker for diagnosis, resulting in a significant decrease in PC-related mortality, diagnosis of PC remains limited. Due to its relatively low specificity, the PCA test is limited by overdiagnosis and prognostic inaccuracy, which in turn results in overtreatment, including surgical intervention, radiation, or ablative focal therapies that can significantly affect patient quality of life. Additionally, although the PSA test broadly correlates with disease outcome at initial diagnosis, its prognostic accuracy is limited if used alone. Therefore, the PSA test is combined with clinical and tumor histological factors for predicting outcomes. Furthermore, to improve PC diagnosis, specific novel biomarkers for PC are required (2–4).

MicroRNAs (miRNAs) are small non-coding RNAs comprising approximately 22 nucleotides that regulate target gene expression by binding to the 3′ untranslated region (UTR) of target mRNAs (5). Several studies have reported the dysregulation of miRNAs in various types of human cancers, including breast cancer (6), lung cancer (7, 8), and PC (9, 10). miRNAs can act as oncogenes or tumor suppressors depending on their target genes. miR-20b-5p suppresses tumorigenesis by targeting cyclin D1 in colon cancer (11), while miR-1226-3p induces apoptosis by downregulating the phosphatidylinositol 3-kinase/protein kinase B pathway in breast cancer (12). These studies have suggested that tumor-suppressor miRNAs are key to understanding the molecular mechanisms underlying cancer progression. More specifically, miR-31-3p has been shown to suppress the progression of cervical cancer (13), while being significantly downregulated in colorectal cancer (14) and medullary thyroid carcinoma (15). However, the function of miR-31-3p in PC remains unclear.

Gamma-aminobutyric acid (GABA) is the main inhibitory neurotransmitter in the vertebrate central nervous system (CNS) of mammals. GABA regulates CNS excitability *via* three classes of receptors. The GABAA and GABAC receptors are ligand-gated ion channels or ionotropic receptors, whereas the GABAB receptor is the G protein-coupled receptor. GABAB receptors are heterodimers composed of GABBR1 and GABBR2 subunits (16). GABA is a known regulator of tumorigenesis. Indeed, several studies have indicated that GABAB receptor activation inhibits cell proliferation and migration in gastric, colon, and malignant cancers. In contrast, other studies have reported that GABAB receptor suppression inhibits cell proliferation and migration in breast cancer metastasis, renal cell carcinoma, and PC metastasis. In particular, reports have shown that GABAB receptors transactivate the epidermal growth factor receptor pathway, resulting in the progression, migration, and invasion of PC and chondrosarcoma. Hence, accumulating evidence has highlighted the role of GABAB receptor in human cancer (2, 17, 18).

In this study, the function, and underlying mechanism of an miRNA screened from those assocaited with the regulation of PC cell line (DU145, PC-3, and LNCap) proliferation, was assessed *in vitro* and *in vivo*. Results show that miR-31-3p functions as a tumor suppressor in PC by directly targeting the GABBR2 receptor.

## 2 Materials and methods

### 2.1 Cell culture and reagents

The human prostate cell line, RWPE-1, was obtained from American Type Culture Collection and cultured in Dulbecco’s modified eagle’s medium (DMEM; 1×; Welgene Inc.) supplemented with 10% fetal bovine serum (FBS, Gibco Thermo Fisher Scientific Inc.) and penicillin-streptomycin (100 U/mL; Gibco Thermo Fisher Scientific Inc.) at 37°C in a humidified incubator containing 5% CO_2_. The human PC cell lines, DU145, PC-3, and LNCap, were obtained from the Korean Cell Line Banker and cultured in Roswell Park Memorial Institute (RPMI) 1640 medium (1×; Welgene Inc.) supplemented with 10% FBS (Gibco Thermo Fisher Scientific Inc.) and penicillin-streptomycin (100 U/mL; Gibco Thermo Fisher Scientific Inc.) at 37°C in a humidified incubator containing 5% CO_2_. The antibodies against p-AKT (cat. no. 4060), AKT (cat. no.4085), p-ERK (cat. no. 4370), p-ERK (cat. no. 4695), and Bak (cat. no. 12105) were purchased from Cell Signaling Technology Inc., while those against BAX (cat. no. sc-53959), β-actin (cat. no. sc-47778), GABBR2 (cat. no. sc-393286), PCNA (cat. no. 25280), p-JNK (cat. no. sc-6254), JNK (cat. no. sc-7345) and BCL-2 (cat. no. sc-7382) were purchased from Santa Cruz Biotechnology Inc.

### 2.2 Selection of candidate growth-inhibiting microRNAs

The miRNA-seq data for PC and normal samples were obtained from the GEO database and analyzed using the Illumina HiSeq system. After downloading the miRNA-seq datasets, the expression data for the remaining miRNAs were log2-transformed. The selected miRNAs were then transfected into DU145, PC-3, and LNCap cell lines to examine their function as cancer cell growth suppressors.

### 2.3 Transfection with RNA oligonucleotides and expression vectors

The miR-31-3p mimic was synthesized by Genolution Pharmaceuticals Inc. RNA duplexes were designed from using sequences (5′- UGC UAU GCC AAC AUA UGC CAU-3’) registered in the miRBase database 22.1 (http://www.mirbase.org). An RNA duplex containing the sequence 5′-CCU CGU GCC GUU CCA UCA GGU AGU U-3′ was used as the control miRNA. Additionally, the pCMV6-Entry plasmid, as well as the expression vector for GABBR2 containing an N-terminal Myc with a pCMV6-Entry backbone (cat. no. RC219404) were purchased from OriGene Technologies, Inc. For functional analyses, DU145, PC-3, and LNCap cells were transfected at 30% confluence with an miRNA mimic at a final concentration of 20 nM and expression vectors (4 μg) using G-fectin (Genolution Pharmaceuticals Inc.) according to the manufacturer’s protocol.

### 2.4 RNA isolation and reverse transcription-quantitative PCR

Total RNA was isolated from the transfected cells or tumor tissues using the TRIzol^®^ reagent (Invitrogen; Thermo Fisher Scientific Inc.) according to the manufacturer’s protocol. To quantify mRNA expression, total RNA (1 μg) was reverse-transcribed into cDNA using Maxime RT PreMix (iNtRON Biotechnology Inc.). The reaction mixtures were incubated at 45°C for 60 min and 95°C for 5 min. qPCR was performed to quantify mRNAs using the AMPIGENE qPCR Green Mix Lo-ROX (Enzo Life Sciences Inc.) and normalized to the level of glyceraldehyde 3-phosphate dehydrogenase (*GAPDH*). The thermocycling reaction conditions were as follows: initial activation at 95°C for 2 min and 40 cycles at 95°C for 5 s and 60°C for 20 s. The sequences of the specific primers for each mRNA were as follows: *GABBR2* forward, 5′-ACCTGTGTATCCTGATCTGC-3’ and reverse, 5′-GGTCTCCCAAGCTAAGAAAC-3’; *CHMP4B* forward, 5′-AGAAGCACGGCACCAAAAAC-3’ and reverse, 5′-GCTGGAACTCGATGGTTGATAAT-3’; *AS3MT* forward, 5′-CTGGGTGGTGCTTTATACTG-3’ and reverse, 5′-TGGTTGGTCCTGTCTTAGAG-3’; and *GAPDH* forward, 5′-GGAGCGAGATCCCTCCAAAAT-3’, and reverse, 5′-GGCTGTTGTCATACTTCTCATGG-3’.

To quantify miRNA expression, total RNA (1 μg) was reverse-transcribed into cDNA using the HB miR multi assay kit SYSTEM I (Heimbiotek Inc.). The reaction mixtures were incubated consecutively at 37°C for 60 min and 95°C for 5 min. qPCR for miRNA quantification was performed using the HB miR multi assay kit SYSTEM I and the data was normalized to the level of *RNU6B*. The thermocycling reaction conditions were as follows: initial activation at 95°C for 15 min and 40 cycles at 95°C for 5 s and 60°C for 40 s. The specific primer sequences for each miRNA were synthesized by Heimbiotek Inc. The expression levels of mRNA and miRNA were calculated according to the 2^-ΔΔCq^ method. Experiments were performed in triplicate and repeated three times.

### 2.5 Determination of cell growth

Cell growth was determined using a cell counting kit-8 (CCK-8) solution according to the manufacturer’s instructions. Briefly, DU145, PC-3, and LNCap cells were transfected with control miRNAs or miR-31-3p in 96-well plates and incubated at 37°C in the presence of 5% CO_2_ for 72 h. After incubation, 10 μL of CCK-8 solution was added, the plates were incubated at 37°C for 2 h, and the absorbance was measured at 450 nm. Experiments were performed in triplicate and repeated three times.

### 2.6 Colony formation assay

DU145, PC-3, and LNCap cells were transfected with control miRNA or miR-31-3p for 48 h. Subsequently, the cells were seeded in 60-mm dishes at a density of 500 cells/dish and were incubated at 37°C in the presence of 5% CO_2_ for 2 weeks. The medium was replaced with fresh growth medium every 4 days. Colonies were counted after staining with a 0.05% crystal violet solution for 30 min. Experiments were performed in triplicate and repeated three times.

### 2.7 Apoptosis analysis

Apoptosis in cultured cells was analyzed using flow cytometry following annexin-FITC/propidium iodide (PI) double staining using an Annexin V-FITC apoptosis detection kit I (BD Biosciences). According to the manufacturer’s protocols, the cells were transfected with control miRNA or miR-31-3p and cultured for 72 h. After the cells were stained, the apoptosis rate was analyzed using a CytoFLEX flow cytometer (Beckman Coulter Life Sciences) and CytExpert software version 2.4 (Beckman Coulter Life Sciences), and the early and late apoptotic cells were enumerated. Experiments were performed in triplicate and repeated three times.

### 2.8 Western blotting

Cells were lysed by incubating in PRO-PREPTM protein extraction solution (iNtRON Biotechnology Inc.) to harvest the proteins. The denatured proteins were separated *via* electrophoresis on 8–12% sodium dodecyl-sulfate polyacrylamide gel and transferred to polyvinylidene difluoride (PVDF) membranes. The PVDF membranes were then blocked in 5% skim milk in Tris-buffered saline (TBS)-Tween 20 (TBST; iNtRON Biotechnology Inc.) at room temperature for 1 h and incubated overnight with specific primary antibodies diluted to 1:500–1:1000 in blocking solution at 4°C. The PVDF membranes were then washed thrice with TBST and incubated with horseradish peroxidase-conjugated anti-mouse IgG or anti-rabbit IgG secondary antibodies diluted 1:4000 in TBST at room temperature for 2 h, followed by detection using a western enhanced chemilumiscence (ECL) substrate (Bio-Rad Laboratories Inc.). Quantification of band intensities was conducted using ImageJ software version 1.52a (National institutes of Health) and normalized to β-actin. Experiments were performed in triplicate and repeated three times.

### 2.9 Sphere formation assay

For sphere formation culture, the cells were incubated in Dulbecco’s modified eagle’s/F12 serum-free medium (Welgene Inc.) supplemented with 2% B-27 (Gibco; Thermo Fisher Scientific Inc.), 20 ng/mL recombinant human epidermal growth factor (Gibco; Thermo Fisher Scientific Inc.), and 20 ng/mL recombinant human fibroblast growth factor basic (Gibco; Thermo Fisher Scientific Inc.) in six-well ultra-low cluster plates (Corning Inc.). Once the diameters of the formed spheres reached 50 µm, the spheroids were transfected with miR-31-3p or control miRNA at a final concentration of 80 nM using G-fectin (Genolution Pharmaceuticals Inc.) according to the manufacturer’s protocol. Four days after transfection, images of the spheres were captured using an Olympus CKX52 microscope (magnification, ×40), and the number and diameter of the spheres were measured in four randomly selected fields. Experiments were performed in triplicate and repeated three times.

### 2.10 Transwell assay

A transwell assay was performed to estimate migration and invasion abilities. Briefly, DU145, PC-3, and LNCap cells transfected with the control miRNA or miR-31-3p were suspended in serum-free RPMI-1640 medium. The suspended cells were reseeded at a density of 10^5^ cells/well onto filters or Matrigel-coated filters in the upper chamber of an 8.0-μm-pore transwell plate (Corning Inc.). After incubation for 72–96 h, the migrated and invaded cells on the lower surface of the filters were fixed in methanol and stained using 0.05% crystal violet solution. The stained cells were counted in four randomly selected fields using an Olympus CKX52 microscope (magnification, ×200). Experiments were performed in triplicate and repeated three times.

### 2.11 Selection of the putative targets of miR-31-3p

Putative binding sites for miR-31-3p were predicted using the miR target prediction programs DIANA-MICROT (http://www.diana.imis.athena-innovation.gr), TargetScan (http://www.targetscan.org), and miRDB (http://www.mirdb.org). The genes predicted using all three algorithms that contained the putative binding sites for miR-31-3p in their 3′-UTRs were selected as targets of miR-31-3p.

### 2.12 Reporter assay

To prepare the reporter constructs, a DNA fragment of the human GABBR2 3′-UTR containing the putative miR-31-3p binding site was cloned into a pGL3UC vector (provided by V.N. Kim, Seoul National University, Republic of Korea). The 3′-UTR sequence of GABBR2 containing the putative binding sites of miR-31-3p was amplified by Macrogen Inc. To generate mutant reporters, nucleotide mutations were introduced into the putative miR-31-3p binding sites using a QuikChange II site-directed mutagenesis kit (Agilent Technologies) according to the manufacturer’s instructions. DU145, PC-3, and LNCap cells were seeded in 24-well plates (10^5^ cells/well) and co-transfected with the reporter plasmid or empty pGL3UC (100 ng), Renilla plasmid (100 ng), control miRNA or miR-31-3p (40 nM) using Lipofectamine^®^ 3000 according to the manufacturer’s protocol. At 48 h after transfection, luciferase activity was measured using a Dual-Luciferase^®^ reporter assay system according to the manufacturer’s protocol (Promega Corporation). Renilla luciferase activity was used to normalize firefly luciferase activity. Experiments were performed in triplicate and repeated three times.

### 2.13 Tumor xenograft experiments

Animal experimental procedures were reviewed and approved by the CHA University Animal Care and Use Committee (No. 210071). Female BALB/c nu/nu mice (4 weeks old) were purchased from Orient Bio Inc. and maintained under specific pathogen-free conditions at 23 ± 1°C with 50 ± 10% humidity and a 12/12-h light/dark cycle. Food and water were provided *ad libitum*. Mice with DU145 cell xenografts were established by subcutaneously injecting 1x10^7^ cells into the left shoulder of 5-week-old mice. miRNA was injected intratumorally when the average tumor size reached 150 mm^3^. According to the manufacturer’s protocol, 10 μg of control miRNA or miR-31-3p was complexed with 1.2 μL *in vivo*-jetPEI^®^ (Polyplus-transfection^®^) in a final injection volume of 50 μL for each mouse. Tumor size was measured twice per week, and the tumor volume was calculated using the following equation: tumor volume (V) 〖mm〗^3=〖(small diameter)〗^2 × (large diameter)× (π/6). Mice were sacrificed using a CO_2_ euthanasia chamber before the tumor volume reached 1,000 mm^3^, with a fill rate of 30% of the chamber volume/min.

For immunohistochemical (IHC) analysis of murine tumor tissues, a ready-to-use IHC/ICC kit (Biovision Inc.) was used according to the manufacture’s protocol. Briefly, tumor tissues were fixed with 3.7% paraformaldehyde at room temperature, embedded in paraffin, deparaffinized, rehydrated and protein-blocked. The slides were then incubated with specific primary antibodies against GABBR2 (1:100, cat. no. sc-393286), PCNA (1:200, cat. no. sc-25280), BCL-2 (1:100, cat. no. sc-7382) and BAX (1:100, cat. no. sc-53959) for 30 min at room temperature. After washing with PBS, the slides were incubated with HRP-anti-mouse IgG polyclonal antibody for 20 min at room temperature and subsequently developed *via* staining with 3,3′-diaminobenzidine for 10 min at room temperature. Images were captured using ZEISS Axioscan 7. Quantification of IHC stained areas was conducted using ImageJ software version 1.52a (National institutes of Health).

### 2.14 Statistical analysis

All data are presented as the mean ± standard deviation, with a P-value < 0.05 considered statistically significant. All statistical analyses were performed using the GraphPad Prism software 5 (GraphPad Software Inc.). Statistical significance between two groups was evaluated by the Mann-Whitney U test. Kruskal–Wallis test with Bonferoni *post hoc* analsysis was used for multiple group comparisons.

## 3 Results

### 3.1 Selection of miR-31-3p as an effective growth inhibitor based on the gene expression omnibus database

**Figure 1:**
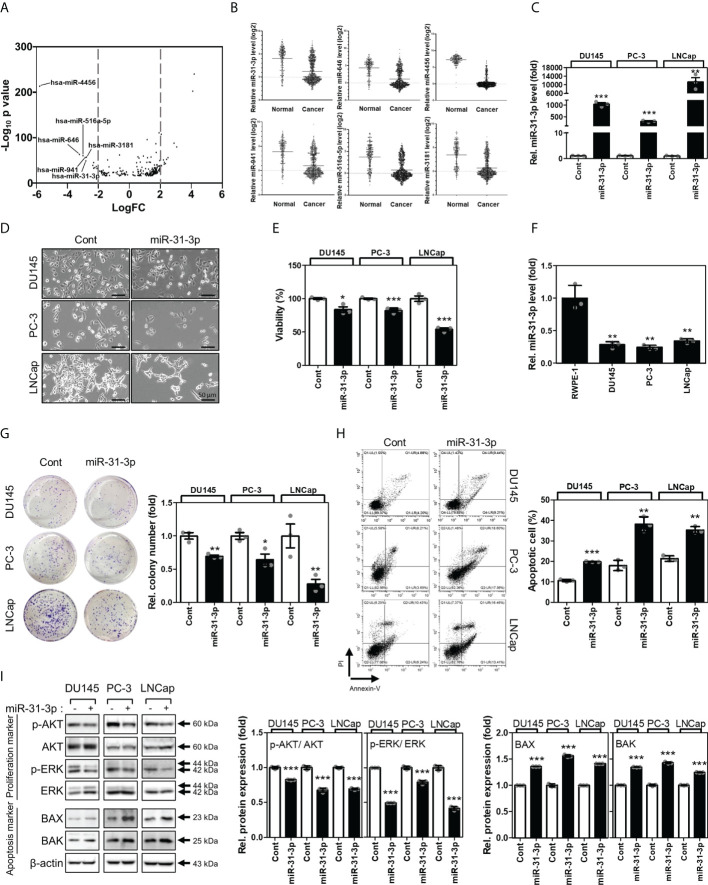
miR-31-3p induces prostate cancer cell apoptosis. **(A)** Volcano plots of the expressions of miRNAs for Gene Expression Omnibus data (GSE112264). The X-axis is the Log2 of miRNA expression (fold change) levels and the Y-axis adjusts the p value as a function of − Log10. **(B)** Relative expression levels of the 6 miRNAs in Gene Expression Omnibus data (GSE112264). **(C)** miR-31-3p expression after transfection with miR-31-3p. An unpaired two-tailed Student’s *t*-test was used to calculate P values. Error bars represent mean ± standard error of the mean (SEM). **P value < 0.01 and ***P value < 0.001 vs. Cont (n = 3). **(D)** Morphology of prostate cancer (PC) cells transfected with the control miRNA or miR-31-3p. **(E)** Growth-inhibiting effects of miR-31-3p. Unpaired two-tailed Student’s *t*-test was used to calculate the P value. Error bars represent mean ± SEM. *P value < 0.05 and ***P value < 0.001 vs. Cont (n = 3). **(F)** Comparison of miR-31-3p expression in normal and cancer cell lines. An unpaired two-tailed Student’s *t*-test was used to calculate P values. Error bars represent mean ± standard error of the mean (SEM). **P value < 0.01 vs. Cont (n = 3). **(G)** Effect of miR-31-3p on colony formation. An unpaired two-tailed Student’s *t-*test was used to calculate P values. Error bars represent mean ± SEM. *P value < 0.05 and **P value < 0.005 vs. Cont (n = 3). **(H)** Effect of miR-31-3p on DU145, PC-3, and LNCap cell apoptosis. An unpaired two-tailed Student’s *t*-test was used to calculate P values. Error bars represent mean ± SEM. **P value < 0.01 and ***P value < 0.001 vs. Cont (n = 3). **(I)** Changes in the expression of proliferation- (p-AKT, AKT, P-ERK, and ERK) and apoptosis-related (Bax and Bak) proteins in DU145, PC-3, and LNCap cells following transfection with control or miR-31-3p. An unpaired two-tailed Student’s *t*-test was used to calculate P values. Error bars represent mean ± SEM. **P value < 0.01 and ***P value < 0.001 vs. Cont (n = 3).

The expression profiles of human miRNAs were analyzed using tissue microarrays of 239 normal serums and 809 PC serums from the GEO datasets (GSE112264). Results show that miR-4456, miR-646, miR-516a-5p, miR-941, miR-31-3p, and miR-3181 were downregulated in PC serums (**Figures 1A, B**). To select the miRNA(s) associated with regulation of cancer cell viability, DU145, PC-3, and LNCap cell lines were transfected with the above-mentioned miRNAs (**Supplementary Figure 1** and **Figure 1C**). Among the six miRNAs analyzed, miR-31-3p was found to inhibit proliferation in all three PC cell lines (**Figures 1D, E**).

### 3.2 miR-31-3p induces apoptosis in PC cells

The functional roles of miR-31-3p in the progression of PC cells were assessed. First, the expression of miR-31-3p between normal prostate (RWPE-1) and cancer cell lines was examined. RWPE-1 cells exhibited significantly higher miR-31-3p expression than the PC cells DU145, PC-3 and LNCap (**Figure 1F**). miR-31-3p transfection also suppressed colony formation in ultralow cluster plates (**Figure 1G**). Furthermore, miR-31-3p transfection significantly increased apoptosis in DU145, PC-3, and LNCap cells (**Figure 1H**). Accordingly, the expression of cell proliferation-associated proteins, namely, p-AKT and p-ERK, was reduced, and that of cell apoptosis-associated proteins (Bax and Bak) was increased (**Figure 1I**).

### 3.3 miR-31-3p suppresses sphere formation, migration, and invasion of PC cells

**Figure 2:**
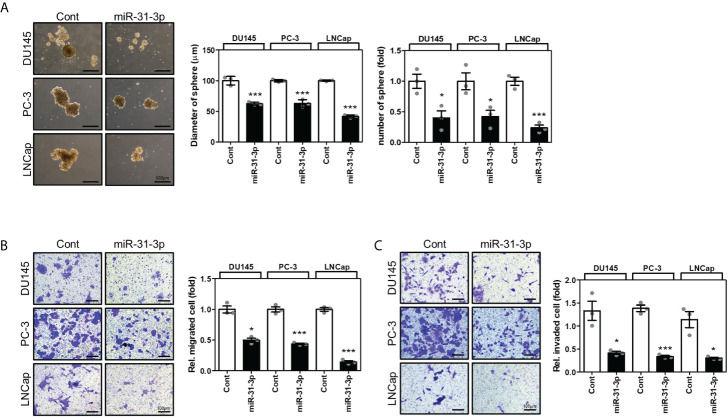
miR-31-3p represses sphere formation, migration, and invasion in prostate cancer (PC) cells. **(A)** miR-31-3p suppresses sphere formation by DU145, PC-3, and LNCap cells. Representative microscopy images are shown. An unpaired two-tailed Student’s *t*-test was used to calculate P values. Error bars represent mean ± standard error of the mean (SEM). *P value < 0.05 and ***P value < 0.001 vs. Cont (n = 3). **(B)** miR-31-3p inhibits DU145, PC-3, and LNCap cell migration. An unpaired two-tailed Student’s *t*-test was used to calculate P values. Error bars represent mean ± SEM. *P value < 0.05 and ***P value < 0.001 vs. Cont (n = 3). **(C)** miR-31-3p inhibits DU145, PC-3, and LNCap cell invasion. An unpaired two-tailed Student’s *t*-test was used to calculate P values. Error bars represent mean ± SEM. *P value < 0.05 and ***P value < 0.001 vs. Cont (n = 3).

The effects of miR-31-3p on the tumorigenesis-related characteristics of PC cells were investigated. First, the ability of miR-31-3p to induce a cancer stem cell (CSC)-like phenotype in PC cells was examined. Sphere culture assays under serum-free conditions have been used to develop CSC-like cells and thus can be used to assess the self-renewal capacity of CSCs. The sphere-forming ability of DU145, PC-3, and LNCap cells in stem cell growth medium was evaluated after transfection with control miRNA or miR-31-3p and incubation for 4 days. miR-31-3p transfection significantly reduced the diameter and number of spheres (**Figure 2A**).

Next, the effect of miR-31-3p on the migratory and invasive capacities of PC cells was determined using a transwell assay. Transfection with miR-31-3p significantly decreased the migratory and invasive abilities of DU145, PC-3, and LNCap cells (**Figures 2B, C**).

### 3.4 miR-31-3p directly targets GABBR2

**Figure 3:**
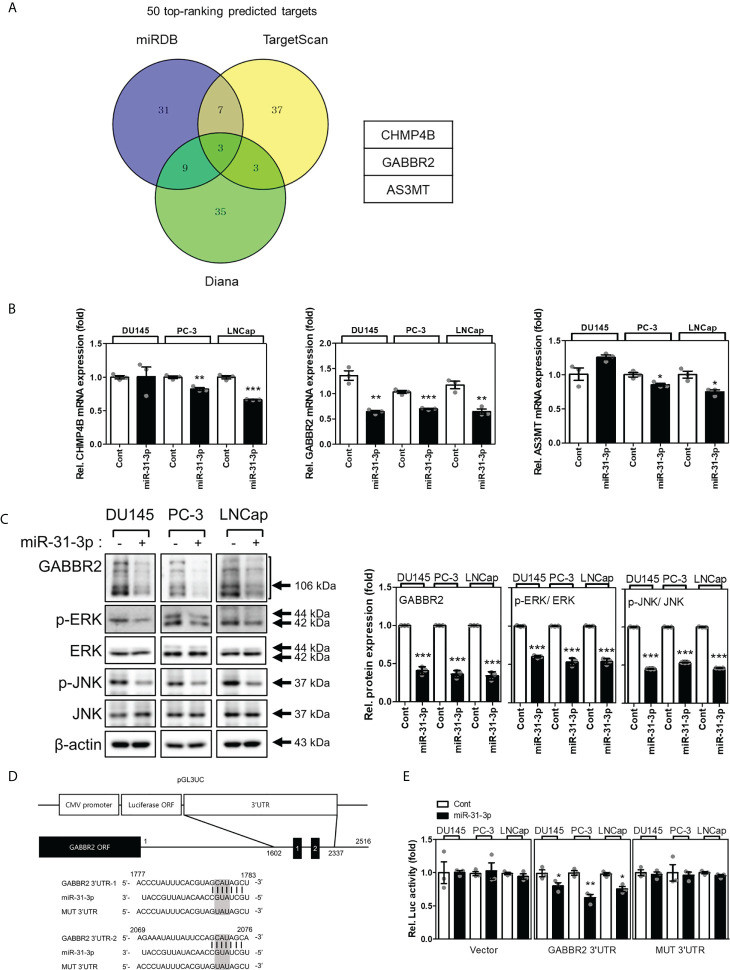
miR-31-3p directly targets GABBR2. **(A)** Venn diagram showing the putative miR-31-3p targets of the top 50 rankings using algorithms to predict miRNA targets. **(B)** miR-31-3p downregulates *GABBR2* mRNA expression. Glyceraldehyde 3-phosphate dehydrogenase was used as the reference gene for normalization. An unpaired two-tailed Student’s *t*-test was used to calculate P values. Error bars represent mean ± standard error of the mean (SEM). *P value < 0.05, **P value < 0.01 and ***P value < 0.001 vs. Cont (n = 3). **(C)** miR-31-3p suppresses the expression of GABBR2, p-ERK, and p-JNK. An unpaired two-tailed Student’s *t-*test was used to calculate P values. Error bars represent mean ± standard error of the mean (SEM). ***P value < 0.001 vs. Cont (n = 3). **(D)** Construction of a reporter including the predicted miR-31-3p target sequence in the 3′-UTR of *GABBR2*. **(E)** Reporter assay indicating direct targeting of the 3′-UTR of *GABBR2* by miR-31-3p. Renilla luciferase activity was used as a reference plasmid to normalize firefly luciferase activity. An unpaired two-tailed Student’s *t*-test was used to calculate P values. Error bars represent mean ± SEM. *P value < 0.05 and **P value < 0.01 vs. Cont (n = 3).

Many candidate proteins were predicted to be targets of miR-31-3p using the miRNA target prediction algorithms, miRDB, TargetScan, and Diana (**Figure 3A**). First, the mRNA expression levels of the three target candidates (i.e., CHMP4B, GABBR2 and AS3MT) were evaluated following transfection of DU145, PC-3, and LNCap cells with miR-31-3p. The mRNA levels of *GABBR2* decreased significantly following transfection of all PC cell lines with miR-31-3p (**Figure 3B**). Similarly, the abundance of GABBR2 protein also decreased significantly in all PC cell lines following transfection. miR-31-3p transfection also reduced the expression of p-ERK and p-JNK, which are downstream targets of GABBR2 (**Figure 3C**) (19).

Based on these results, we investigated whether GABBR2 was a direct target of miR-31-3p using a reporter assay. Two putative miR-31-3p-binding sites that were complementary to the seed region of miR-31-3p were found in the 3′-UTR of GABBR2. The putative miR-31-3p-binding sequence was cloned into pGL3UC, a modified pGL3 reporter vector (**Figure 3D**). Luciferase activity was measured after co-transfection of PC cells with reporter plasmid/empty pGL3UC, Renilla vector, and miRNA. Co-transfection with miR-31-3p significantly decreased the relative luciferase activity of the GABBR2 3′-UTR reporter. In contrast, the luciferase activity of mutant reporters (MUT 3′-UTR) was not decreased after co-transfection with miR-31-3p (**Figure 3E**). These results indicated that GABBR2 was the direct target of miR-31-3p.

### 3.5 miR-31-3p-induced tumor suppressive functions are generated through GABBR2 downregulation

**Figure 4:**
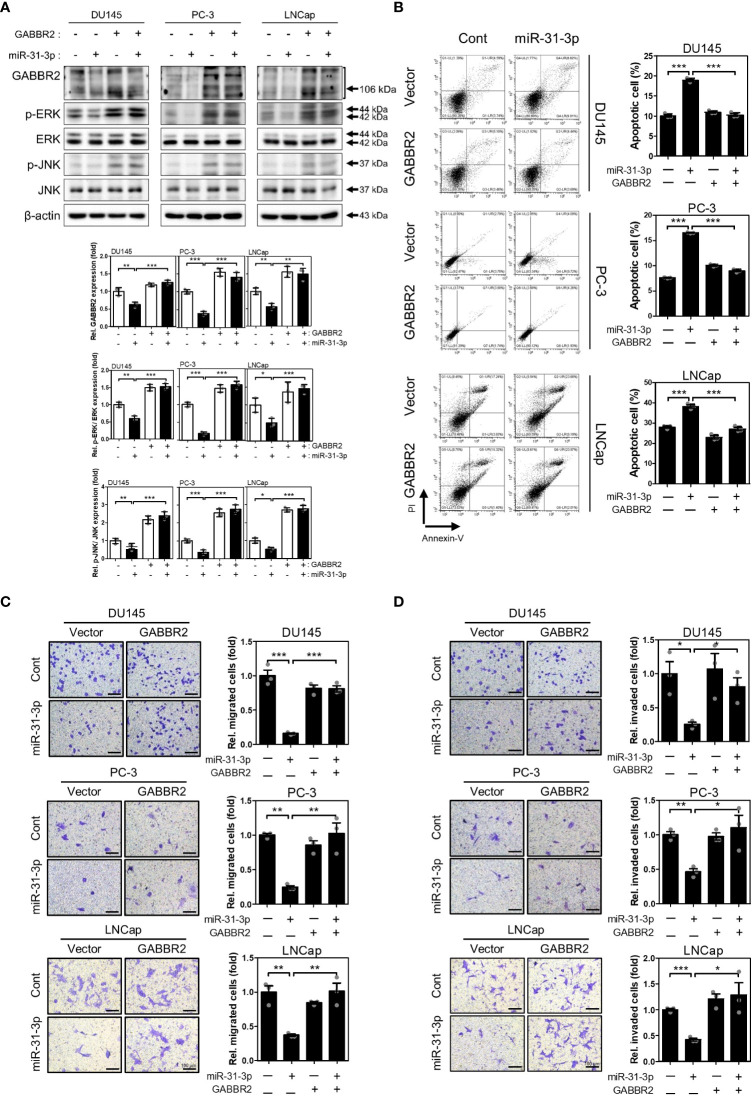
miR-31-3p-induced tumor suppressive functions are associated with downregulation of GABBR2. **(A)** Restoration of GABBR2, p-ERK and p-JNK protein levels after ectopic expression of Myc-DDK-tagged-GABBR2. DU145, PC-3 and LNCap cells were transfected with a mixture of miR-31-3p (20 nM) and GABBR2 expression vector (4 μg). An unpaired two-tailed Student’s t-test was used to calculate P values. Error bars represent mean ± SEM. *P value < 0.05, **P value < 0.01 and ***P value < 0.001 vs. Cont (n = 3). **(B)** Ectopic expression of GABBR2 suppresses miR-31-3p-induced apoptosis of DU145, PC-3, and LNCap cells. An unpaired two-tailed Student’s t-test was used to calculate P values. Error bars represent mean ± SEM. ***P value < 0.001 vs. Cont (n = 3). **(C, D)** Ectopic expression of GABBR2 restores miR-31-3p-mediated inhibition of PC cell migration and invasion. An unpaired two-tailed Student’s t-test was used to calculate P values. Error bars represent mean ± SEM. *P value < 0.05, **P value < 0.01 and ***P value < 0.001 vs. Cont (n = 3).

To validate the significance of directly targeting GABBR2, we evaluated whether redistribution of GABBR2 expression inhibits the effects of miR-31-3p transfection. Ectopic expression of GABBR2, from a Myc-DDK-tagged-GABBR2 expression vector, co-transfected with miR-31-3p, restorated GABBR2, p-ERK and p-JNK protein levels induced by miR-31-3p (**Figure 4A**). Furthermore, ectopic expression of GABBR2 significally suppressed miR-31-3p-induced apoptosis (**Figure 4B**). Additionally, the suppressed migration and invasion of cells following miR-31-3p transfection, were restored to those of control by co-expression of the GABBR2 protein (**Figures 4C, D**). These results indicate that the tumor suppressive functions of miR-31-3p are assocated with direct downregulation of GABBR2.

### 3.6 miR-31-3p inhibits tumor growth in mouse xenografts

**Figure 5:**
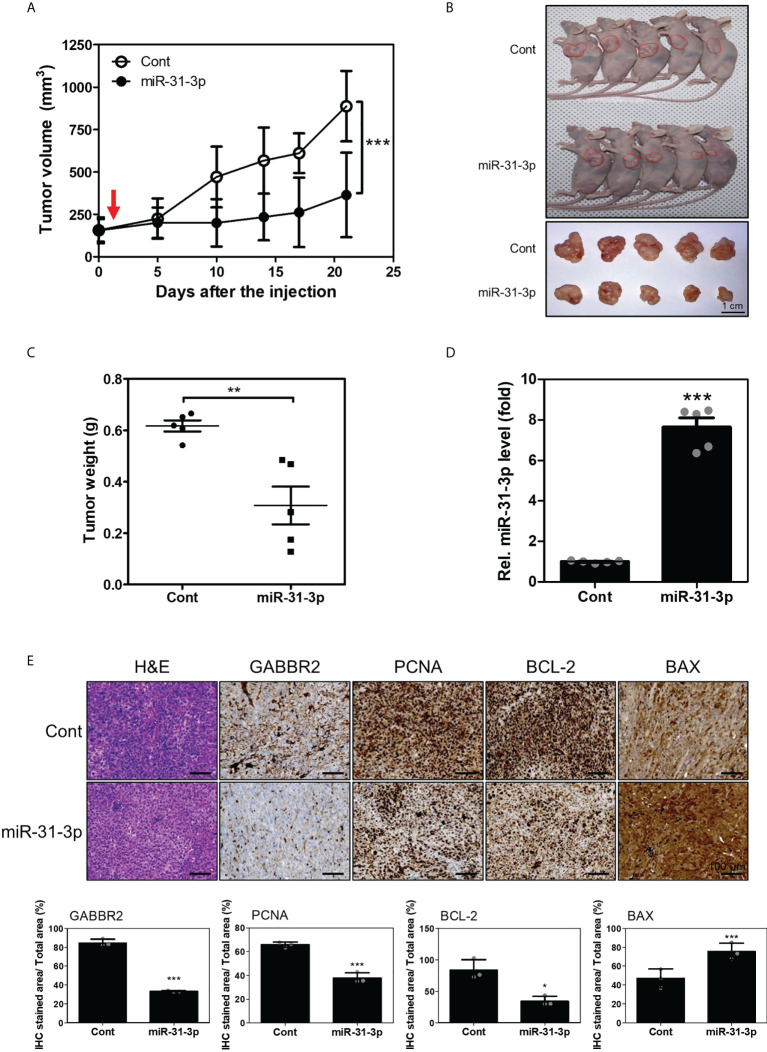
miR-31-3p suppresses *in vivo* tumor growth rate. **(A)** miR-31-3p suppresses tumor growth rate in mouse DU145 cell xenografts (n = 5 per group). An unpaired two-tailed Student’s *t*-test was used to calculate P values. Error bars represent mean ± standard error of the mean. *P value < 0.05 and ***P value < 0.001 vs. Cont (n = 5). **(B)** Representative images of tumors on the shoulders of mice after tumor resection. **(C)** Comparison of tumor weight distributions. An unpaired two-tailed Student’s t-test was used to calculate P values. Error bars represent mean ± standard error of the mean (SEM). **P value < 0.005 vs. Cont (n = 5). **(D)** Confirmation of miR-31-3p induction in tumors of mice transfected with control miRNA or miR-31-3p. RNU6B was used as the reference gene for normalization. An unpaired two-tailed Student’s *t*-test was used to calculate P values. Error bars represent mean ± standard error of the mean (SEM). ***P value < 0.001 vs. Cont (n = 5). **(E)** Immunohistochemical analysis of tumor tissue sections to determine the expression of GABBR2 and related proteins. An unpaired two-tailed Student’s *t*-test was used to calculate P values. Error bars represent mean ± standard error of the mean (SEM). *P value < 0.05 and ***P value < 0.001 vs. Cont (n = 3).

To estimate the *in vivo* tumor-suppressive effects of miR-31-3p, changes in tumor volume in mouse xenografts after miR-31-3p delivery were measured. Tumors established using DU145 cells were injected with control miRNA or miR-31-3p using a mixture of *in vivo* transfection reagents. The growth rate of the miR-31-3p-injected tumors was significantly lower than that of the control miRNA-injected tumors (**Figure 5A**). Additionally, tumor size and weight of miR-31-3p-injected mice were significantly lower than those of control-injected mice at the endpoint (**Figures 5B, C**). The expression of miR-31-3p was also increased in the dissected tumors of mice transfected with miR-31-3p (**Figure 5D**). Immunohistochemistry showed that the intratumoral injection of miR-31-3p decreased the expression of GABBR2, proliferating cell nuclear antigen (PCNA) and BCL2 apoptosis regulator (Bcl-2), a cell proliferation-associated and anti-apoptosis-associated protein, respectively, while increasing the expression of BCL2 associated X-apoptosis regulator (BAX), a cell apoptosis-associated protein (**Figure 5E**). These results suggested that miR-31-3p functioned as a tumor suppressor *in vivo* and may serve as an anticancer miRNA.

## 4 Discussion

The increase in knowledge about miRNA biology has ushered in a new perspective in the development of innovative chemotherapeutics. Over the past decade, numerous studies have shown that miRNAs are abnormally expressed in various types of cancer (20–24), and that they can act as tumor suppressors or oncogenes (25). Hence, understanding the role of miRNAs may contribute to the identification of new potential biomarkers for cancer diagnosis. Recently, the use of synthetic miRNA mimics to inhibit tumor growth, or miRNA inhibitors to suppress the function of oncogenic miRNAs has emerged as the main approach for developing miRNA therapeutics (26). In fact, in 2021, a therapeutic molecule, MRX44, targeting miR-34a, was in a phase 1 clinical trial for the treatment of different types of cancers. Meanwhile, in the current study, miR-31-3p was found to inhibit the growth of PC *in vitro* and to exhibit anti-tumor effects in a mouse xenograft model that was intratumorally administered a miR-31-3p mimic. These findings suggest that miR-31-3p can be used as a potential tumor suppressive miRNA in patients.

Several targets of miR-31-3p were analyzed to identify the genes responsible for the growth-inhibiting action of miR-31-3p. Among the identified targets was GABBR2, a component of GABAB receptors, that required for normal receptor functioning (27). Within the heterodimeric GABA receptor, GABBR2 mediates coupling to G proteins (28). Although the role of GABBR2 in cancer is not yet understood, several reports have revealed its aberrant expression in cancer. Stein and colleagues first reported the possible role of GABBR2 in cancer progression (29). Similarly, Schulten and colleagues reported that GABBR2 was one of the most significantly upregulated genes in the follicular variant of papillary thyroid carcinoma (30). Furthermore, GABBR2 level increased in non-small cell lung cancer, and patients with high expression of GABBR2 showed better prognosis (27). Thus, targeting GABBR2 is a plausible strategy for the development of chemotherapeutics. Our results showed that miR-31-3p significantly inhibits the proliferation, invasion, and spheroid growth of PC. Moreover, miR-31-3p directly targets GABBR2 causing its subsequent downregulation *in vitro* and *in vivo*. These data indicated that miR-31-3p has a tumor-suppressive role by modulating the expression of GABBR2. However, as the role of GABBR2 in cancer remains unclear, additional mechanisms by which miR-31-3p inhibits cancer growth following GABBR2 overexpression should be further investigated.

Since 1941, androgen deprivation therapy (ADT) has been the first-line treatment for patients diagnosed with PC for the first time (31). ADT also represents the key therapeutic strategy for patients with aggressive PC; however, many treated tumors relapse and eventually progress to castration-resistant PC (32). In accordance with this view, previous studies have suggested an intimate relationship between CSCs and treatment failure (33, 34). CSCs reside within the tumor microenvironment and harbor unique metabolic pathways (33). They possess self-renewal capacity and mediate tumor initiation and propagation (35). Reports have shown that CSCs exhibit resistance to anticancer therapies, thereby contributing to recurrence (33, 34). Having demonstrated the role of CSCs in cancer therapy failure, a few studies have speculated a connection between CSCs and PC. For instance, O’Reilly et al. reported that hypoxia-induced CSC enrichment promotes resistance to ADT in PC (36). This finding is further supported by the results of Jeter et al. who showed that NANOG, a pluripotency-related gene, promotes CSC characteristics and resistance of PC to androgen deprivation (37). Herein, we demonstrated the suppressive effects of miR-31-3p on CSCs, implying that miR-31-3p may be developed as a potent miRNA therapeutic for overcoming relapse and malignancy in current therapies.

In summary, we identified miR-31-3p as a potential tumor suppressor miRNA that inhibited proliferation, CSC features, and invasion/metastasis of PC cells *in vitro*. miR-31-3p directly targets GABBR2, indicating that miR-31-3p exerts anti-cancer effects *via* regulation of GABBR2 expression. Additionally, miR-31-3p exerts tumor-suppressive effects in a mouse xenograft model. Taken together, these results suggest that miR-31-3p is a novel tumor suppressor miRNA that purges PC of proliferative activity.

## Data availability statement

Publicly available datasets were analyzed in this study. This data can be found here: https://www.ncbi.nlm.nih.gov/geo/query/acc.cgi?acc=GSE112264.

## Ethics statement

The animal study was reviewed and approved by CHA University Animal Care and Use Committee (No. 210071).

## Author contributions

SC: Project administration, data curation, formal analysis, writing original draft. SL: Project administration, data curation, formal analysis, writing original draft. Y-HH: Conceptualization, data curation. JC: Data curation, formal analysis. IK: Data curation, formal analysis. JL: Data curation. H-JA: Conceptualization, funding acquisition, investigation, methodology, manuscript review and editing. All authors contributed to the article and approved the submitted version.

## Funding

This research was supported by Basic Science Research Program through the National Research Foundation of Korea(NRF) funded by the Ministry of Education(No. 2020R1I1A1A01073917).

## Acknowledgments

The authors thank the members in the lab for their helpful comments, suggestions, and assistance with animal experiments.

## Conflict of interest

The authors declare that the research was conducted in the absence of any commercial or financial relationships that could be construed as a potential conflict of interest.

## Publisher’s note

All claims expressed in this article are solely those of the authors and do not necessarily represent those of their affiliated organizations, or those of the publisher, the editors and the reviewers. Any product that may be evaluated in this article, or claim that may be made by its manufacturer, is not guaranteed or endorsed by the publisher.

## Supplementary material

Supplementary Figure 1 Functional screening for miRNAs regulating PC cells growth. **(A)** miRNAs expression after transfection with indicated miRNA. Total RNA was prepared from transfected cells and indicated miRNA levels were analyzed by real-time polymerase chain reaction. RNU6B was used as the reference gene for normalization. An unpaired two-tailed Student’s t-test was used to calculate P values. Error bars represent mean ± standard error of the mean (SEM). ***P value < 0.001 vs. Cont (n = 3). **(B)** Representative image of morphology of PC cells transfected with the control miRNA, miR-4456, miR-646, miR-516a-5p, miR-941, miR-31-3p or miR-3181. **(C)** Identification of growth-inhibiting effects of miRNAs. Cell viability in cultured cells transfected with control miRNA, miR-4456, miR-646, miR-516a-5p, miR-941, miR-31-3p or miR-3181 was measured using Cell Counting Kit-8 at 72 h. Unpaired two-tailed Student’s t-test was used to calculate the P value. Error bars represent mean ± SEM. **P value < 0.01 and ***P value < 0.001 vs. Cont (n= 3).
