# Corrigendum: miR-31-3p functions as a tumor suppressor by directly targeting GABBR2 in prostate cancer

**DOI:** 10.3389/fonc.2024.1395244

**Published:** 2024-03-18

**Authors:** Sujin Choi, Soonchul Lee, Young-Hoon Han, Junwon Choi, Isaac Kim, Jusung Lee, Hyun-Ju An

**Affiliations:** ^1^ Department of Orthopaedic Surgery, CHA Bundang Medical Center, CHA University School of Medicine, Pangyo-ro, Republic of Korea; ^2^ Division of Radiation Cancer Research, Korea Institute of Radiological and Medical Sciences, Seoul, Republic of Korea; ^3^ Department of Molecular Science and Technology, Ajou University, Yeongtong-gu, Republic of Korea; ^4^ Department of General Surgery, CHA Bundang Medical Center, CHA University School of Medicine, Pangyo-ro, Republic of Korea

**Keywords:** miR-31-3p, GABBR2, prostate cancer, miRNA, tumor suppressor gene

In the published article, there was a mistake in **Figure 4** as published. The image of LNCap co-transfected with empty vector and control miRNA in Panel C was mistakenly mixed up with the image of the LNCap co-transfected with GABBR2 vector and miR-31-3p in Panel D. The corrected **Figure 4** appears below.

**Figure 4:**
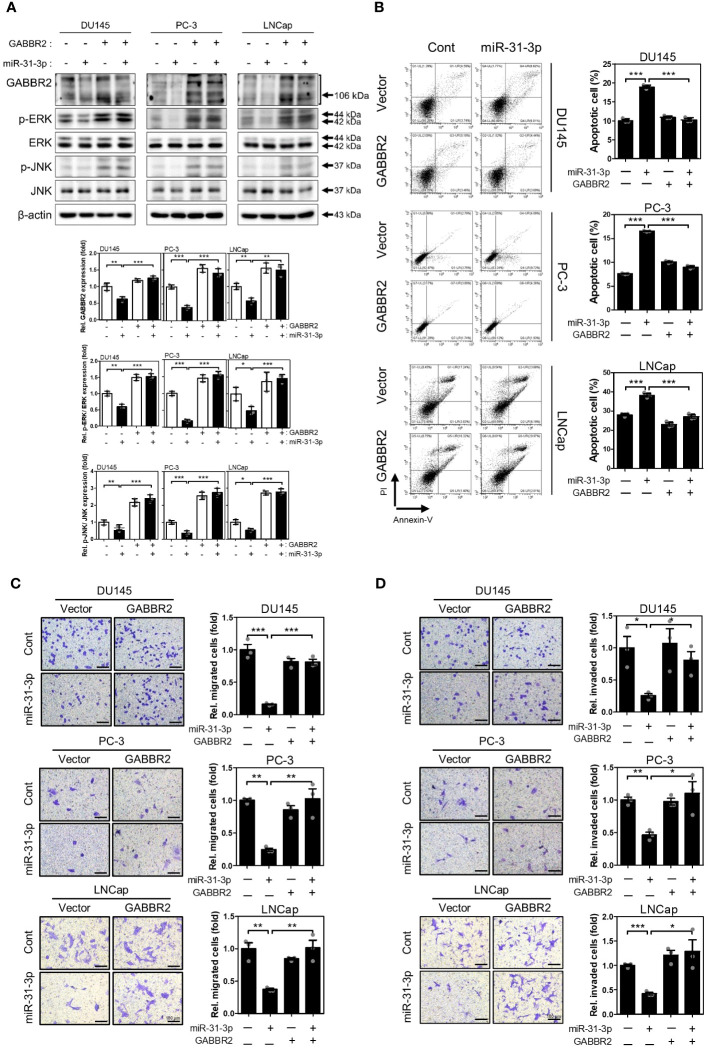
miR-31-3p-induced tumor suppressive functions are associated with downregulation of GABBR2. **(A)** Restoration of GABBR2, p-ERK and p-JNK protein levels after ectopic expression of Myc-DDK-tagged-GABBR2. DU145, PC-3 and LNCap cells were transfected with a mixture of miR-31-3p (20 nM) and GABBR2 expression vector (4 μg). An unpaired two-tailed Student’s t-test was used to calculate P values. Error bars represent mean ± SEM. *P value < 0.05, **P value < 0.01 and ***P value < 0.001 vs. Cont (n = 3). **(B)** Ectopic expression of GABBR2 suppresses miR-31-3p-induced apoptosis of DU145, PC-3, and LNCap cells. An unpaired two-tailed Student’s t-test was used to calculate P values. Error bars represent mean ± SEM. ***P value < 0.001 vs. Cont (n = 3). **(C, D)** Ectopic expression of GABBR2 restores miR-31-3p-mediated inhibition of PC cell migration and invasion. An unpaired two-tailed Student’s t-test was used to calculate P values. Error bars represent mean ± SEM. *P value < 0.05, **P value < 0.01 and ***P value < 0.001 vs. Cont (n = 3).

The authors apologize for this error and state that this does not change the scientific conclusions of the article in any way. The original article has been updated.

