# Supplementary material for: miR-31-3p functions as a tumor suppressor by directly targeting GABBR2 in prostate cancer
Source: Front Oncol. 2022 Aug 18;12:945057. doi: 10.3389/fonc.2022.945057 (PMC9434366; doi:10.3389/fonc.2022.945057)

Supplementary materials.

miR-31-3p Functions as A Tumor Suppressor by Directly Targeting GABBR2 in Prostate Cancer

Sujin Choi[1†], Soonchul Lee[1†], Young-Hoon Han[2], Junwon Choi[3], Isaac Kim[4], Jusung Lee[1], Hyun-Ju An[1*]

[1] Department of Orthopaedic Surgery, CHA Bundang Medical Center, CHA University School of Medicine, 335 Pangyo-ro, Bundang-gu, Gyeonggi-do, Republic of Korea, Zip code: 13488

[2] Division of Radiation Cancer Research, Korea Institute of Radiological and Medical Sciences, 75 Nowon-ro, Nowon-gu, Seoul, Republic of Korea, Zip code: 01812

[3] Department of Molecular Science and Technology, Ajou University, 206 World cup-ro, Yeongtong-gu, Gyeonggi-do, Republic of Korea, Zip code: 16499

[4] Department of General Surgery, CHA Bundang Medical Center, CHA University School of Medicine, 335 Pangyo-ro, Bundang-gu, Gyeonggi-do, Republic of Korea, Zip code: 13488

*** Correspondence:**
Hyun-Ju An
yks486ahj@naver.com

[†]These authors contributed equally to this work.

Supplementary Fig. S1. Western blot of cell lysate in figure. (a) Western blot bands of figure 1I. (b) Western blot bands of figure 3C. (c) Western blot bands of figure 4A.

Supplementary Fig. S1.

(a)

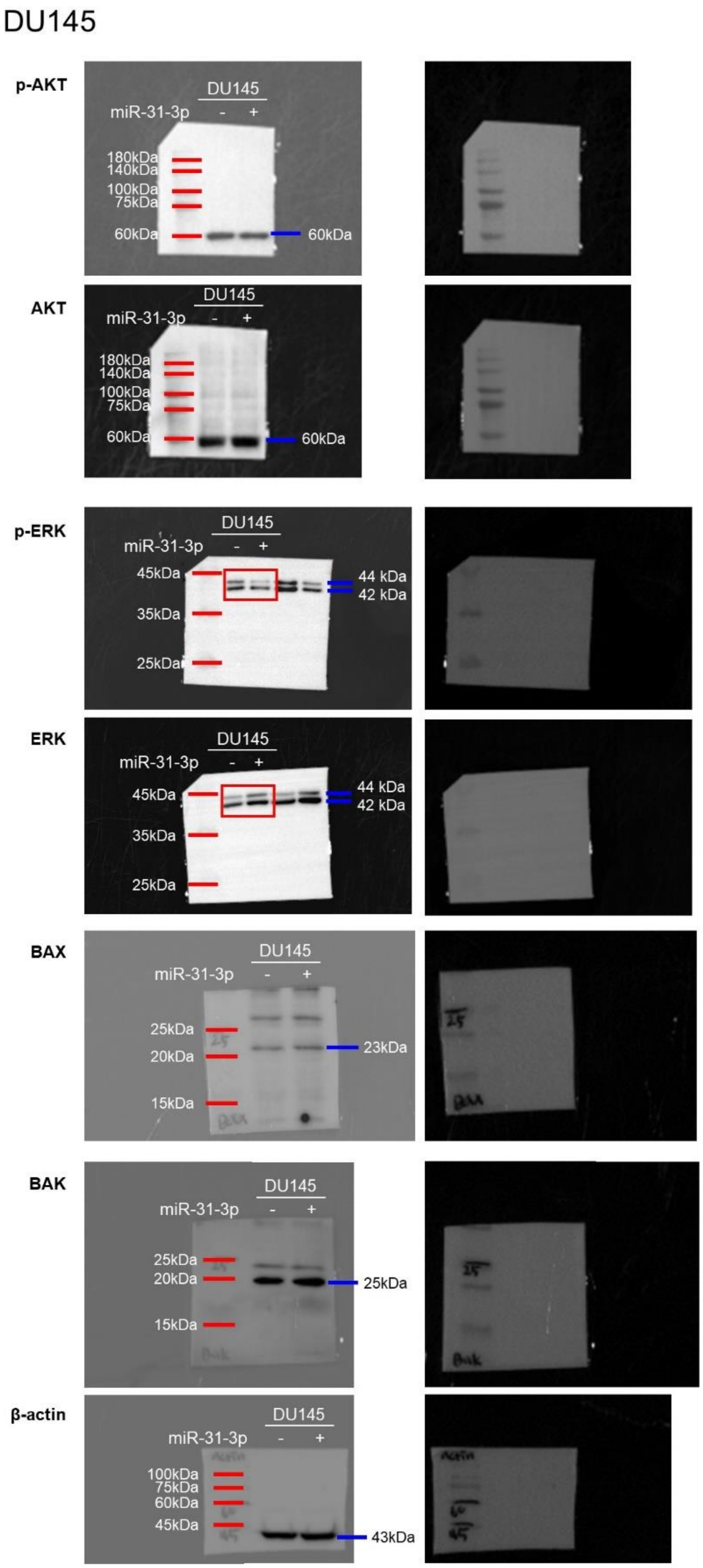

PC-3

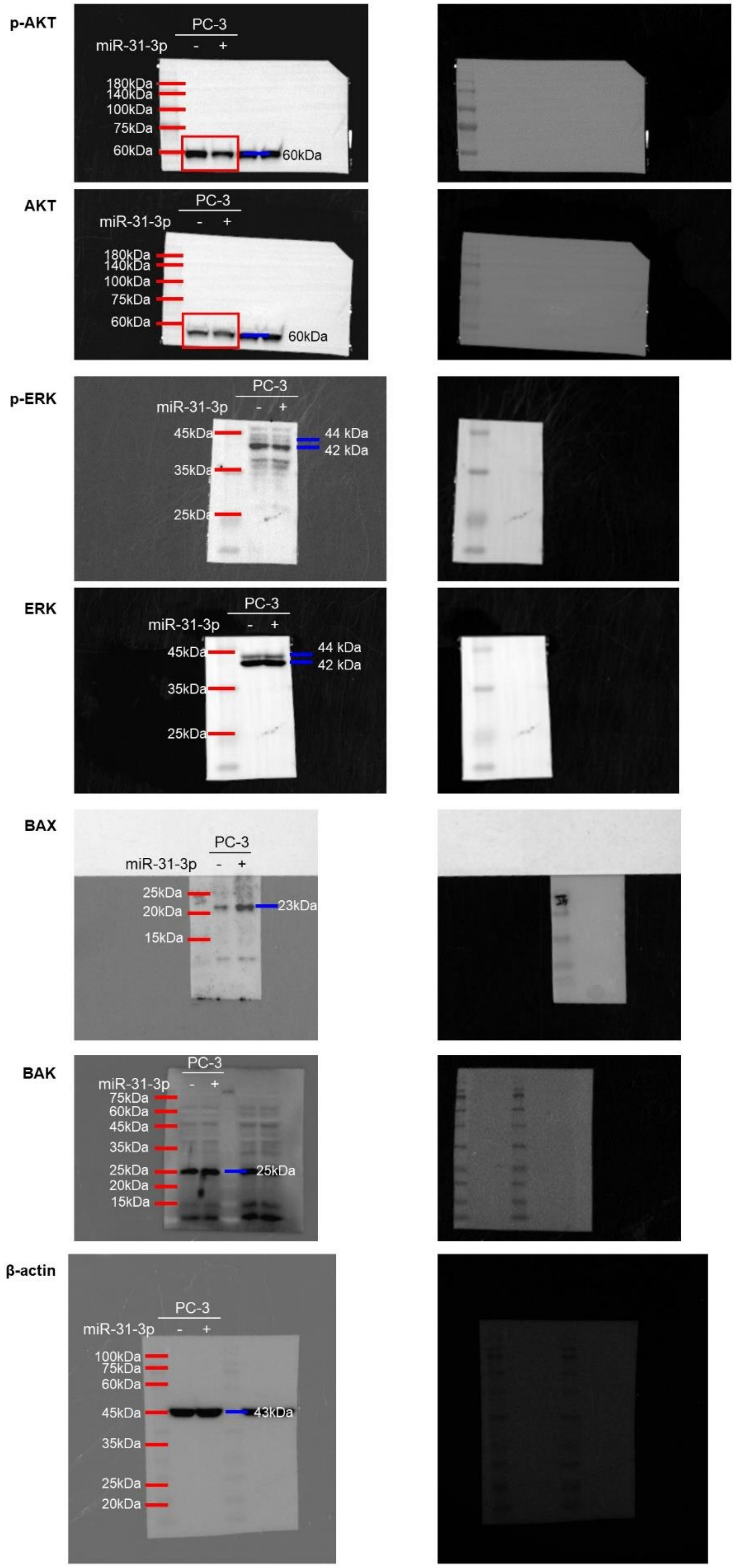
p-AKT
PC-3
miR-31-3p - +
180kDa
140kDa
100kDa
75kDa
60kDa
60kDa
AKT
PC-3
miR-31-3p - +
180kDa
140kDa
100kDa
75kDa
60kDa
60kDa
p-ERK
PC-3
miR-31-3p - +
45kDa
35kDa
25kDa
44 kDa
42 kDa
ERK
PC-3
miR-31-3p - +
45kDa
35kDa
25kDa
44 kDa
42 kDa
BAX
PC-3
miR-31-3p - +
25kDa
20kDa
15kDa
23kDa
BAK
PC-3
miR-31-3p - +
75kDa
60kDa
45kDa
35kDa
25kDa
20kDa
15kDa
25kDa
β-actin
PC-3
miR-31-3p - +
100kDa
75kDa
60kDa
45kDa
35kDa
25kDa
20kDa
43kDa

LNCap

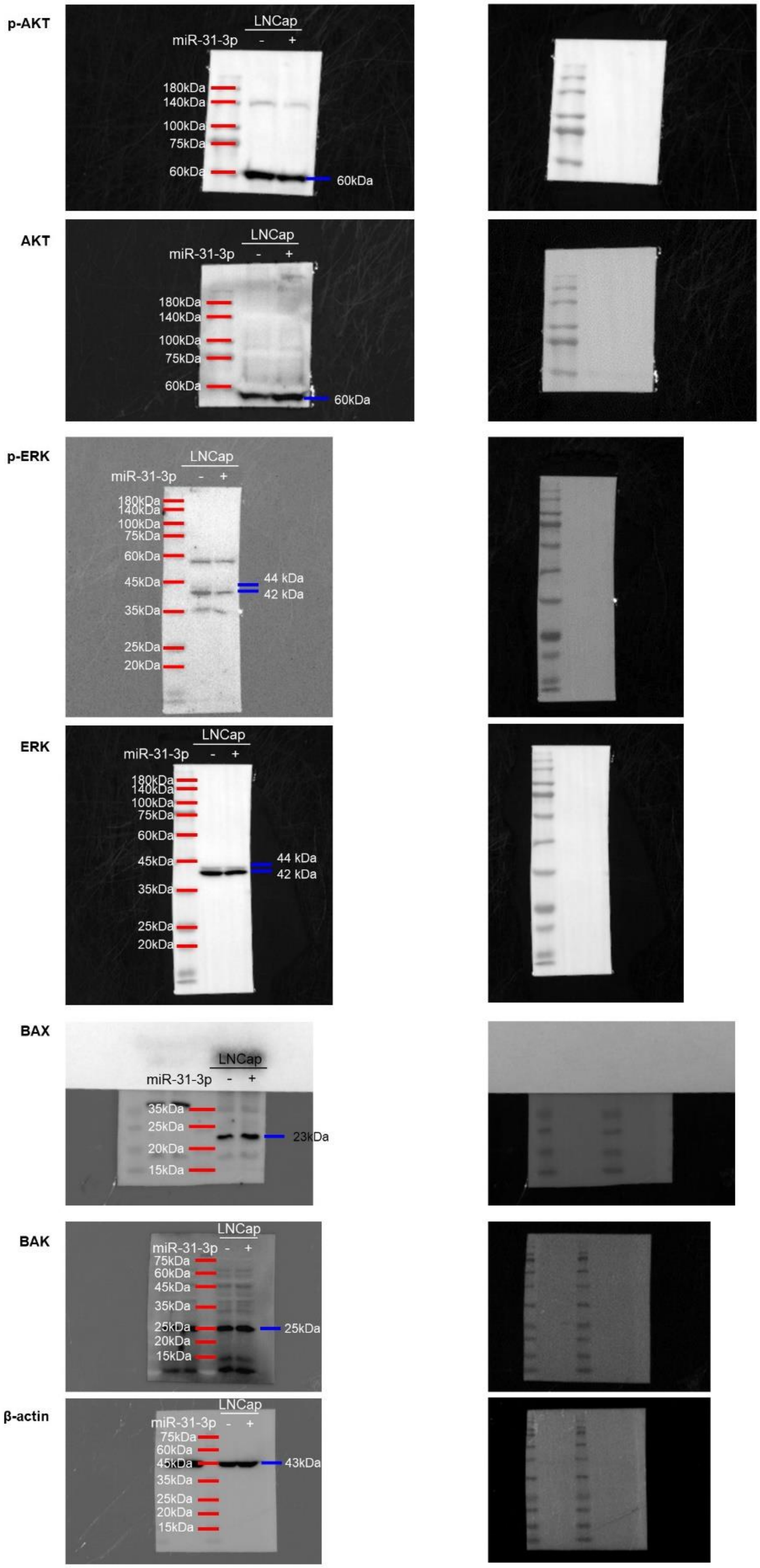
p-AKT
LNCap
miR-31-3p - +
180kDa
140kDa
100kDa
75kDa
60kDa
60kDa
AKT
LNCap
miR-31-3p - +
180kDa
140kDa
100kDa
75kDa
60kDa
60kDa
p-ERK
LNCap
miR-31-3p - +
180kDa
140kDa
100kDa
75kDa
60kDa
45kDa
35kDa
25kDa
20kDa
44 kDa
42 kDa
ERK
LNCap
miR-31-3p - +
180kDa
140kDa
100kDa
75kDa
60kDa
45kDa
35kDa
25kDa
20kDa
44 kDa
42 kDa
BAX
LNCap
miR-31-3p - +
35kDa
25kDa
20kDa
15kDa
23kDa
BAK
LNCap
miR-31-3p - +
75kDa
60kDa
45kDa
35kDa
25kDa
20kDa
15kDa
25kDa
β-actin
LNCap
miR-31-3p - +
75kDa
60kDa
45kDa
35kDa
25kDa
20kDa
15kDa
43kDa

(b)

DU145

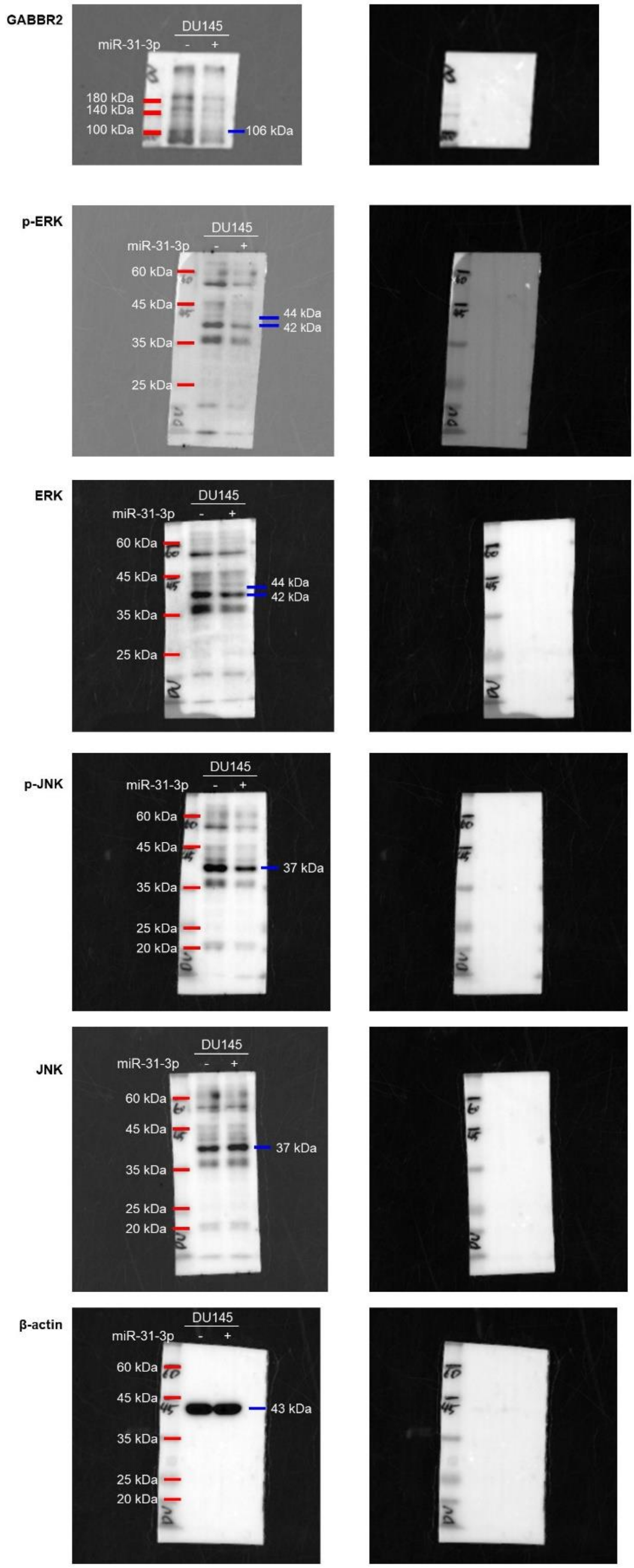
GABBR2
DU145
miR-31-3p - +
180 kDa
140 kDa
100 kDa
106 kDa
p-ERK
DU145
miR-31-3p - +
60 kDa
45 kDa
35 kDa
25 kDa
44 kDa
42 kDa
ERK
DU145
miR-31-3p - +
60 kDa
45 kDa
35 kDa
25 kDa
44 kDa
42 kDa
p-JNK
DU145
miR-31-3p - +
60 kDa
45 kDa
35 kDa
25 kDa
20 kDa
37 kDa
JNK
DU145
miR-31-3p - +
60 kDa
45 kDa
35 kDa
25 kDa
20 kDa
37 kDa
β-actin
DU145
miR-31-3p - +
60 kDa
45 kDa
35 kDa
25 kDa
20 kDa
43 kDa

PC-3

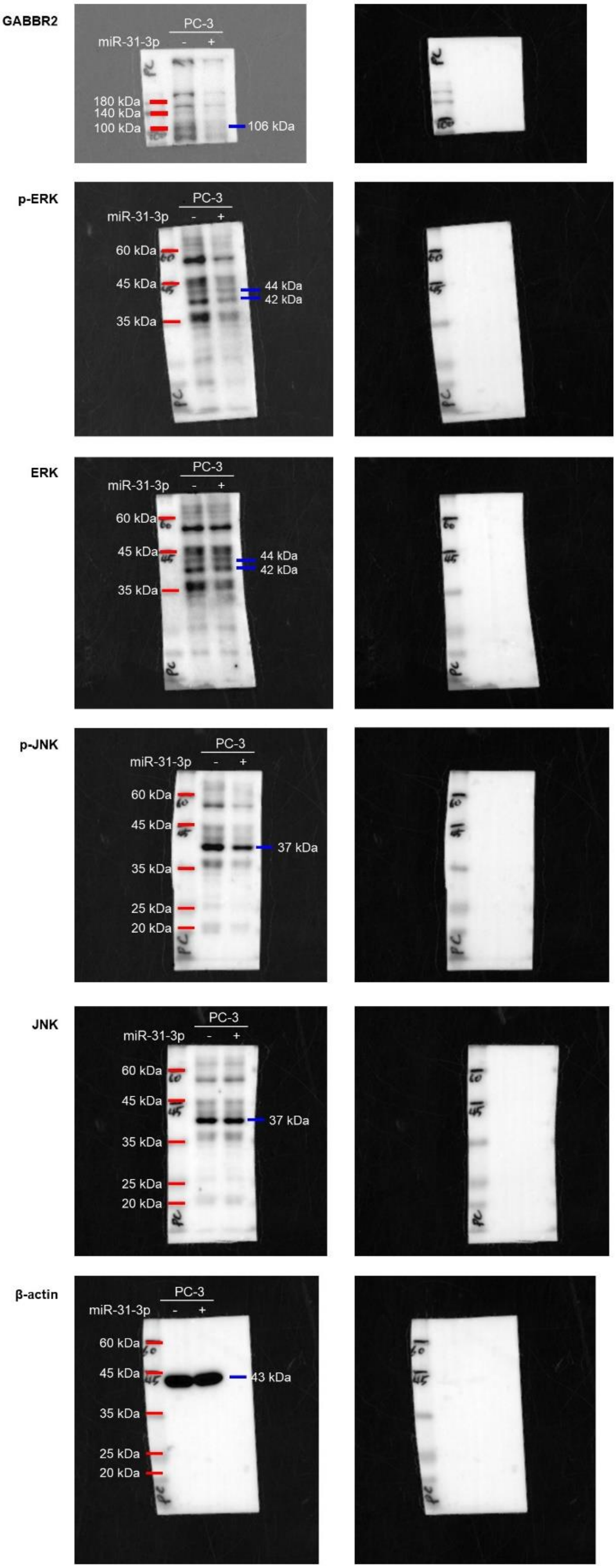
GABBR2
PC-3
miR-31-3p - +
180 kDa
140 kDa
100 kDa
106 kDa
p-ERK
PC-3
miR-31-3p - +
60 kDa
45 kDa
35 kDa
44 kDa
42 kDa
ERK
PC-3
miR-31-3p - +
60 kDa
45 kDa
35 kDa
44 kDa
42 kDa
p-JNK
PC-3
miR-31-3p - +
60 kDa
45 kDa
35 kDa
25 kDa
20 kDa
37 kDa
JNK
PC-3
miR-31-3p - +
60 kDa
45 kDa
35 kDa
25 kDa
20 kDa
37 kDa
β-actin
PC-3
miR-31-3p - +
60 kDa
45 kDa
35 kDa
25 kDa
20 kDa
43 kDa

LNCap

GABBR2

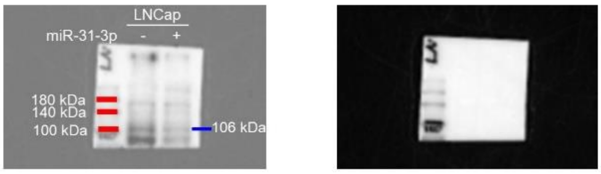

p-ERK

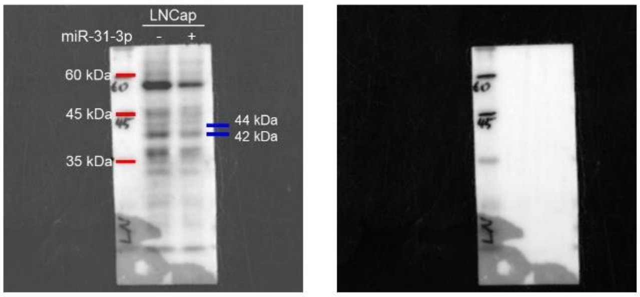

ERK

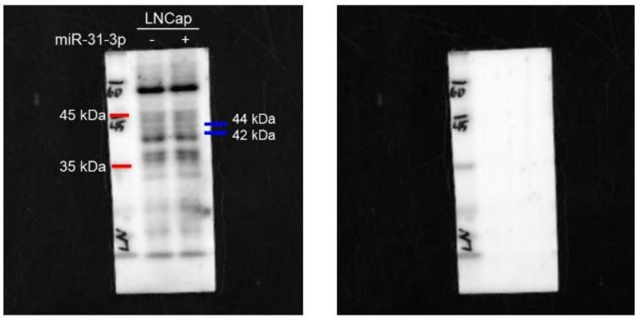

p-JNK

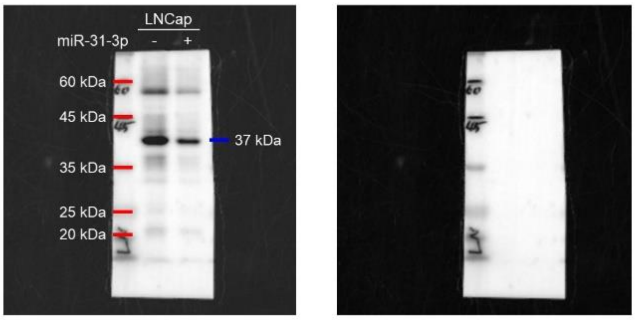

JNK

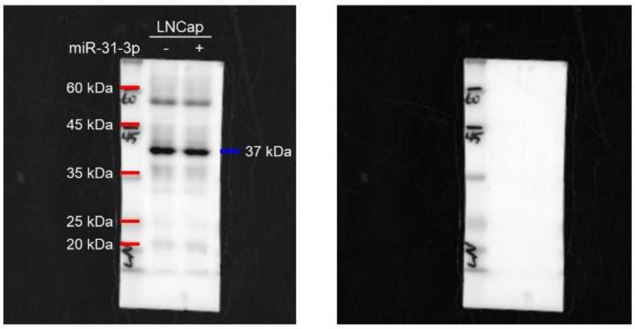

β-actin

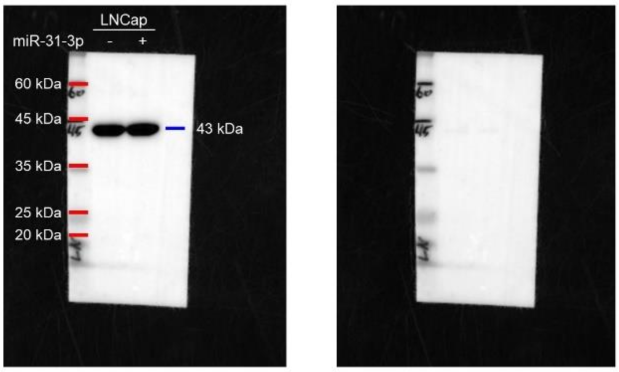

(c)

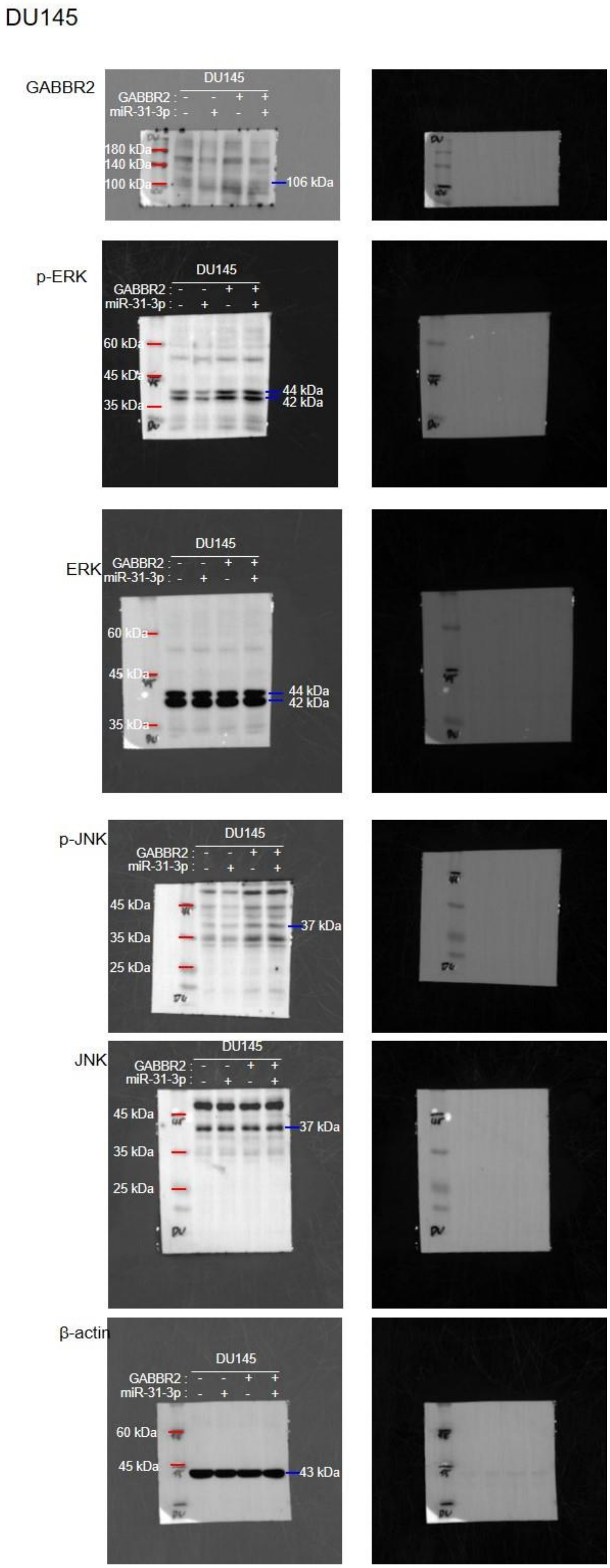
DU145
GABBR2
DU145
GABBR2 : - - + +
miR-31-3p : - + - +
180 kDa
140 kDa
100 kDa
106 kDa
p-ERK
60 kDa
45 kDa
35 kDa
44 kDa
42 kDa
ERK
p-JNK
25 kDa
37 kDa
JNK
β-actin
43 kDa

PC-3

GABBR2

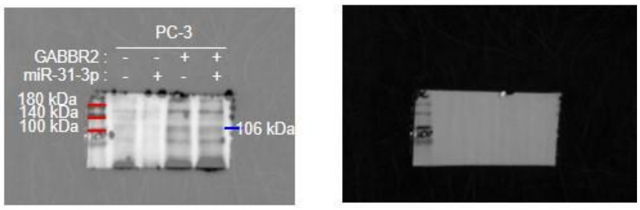

p-ERK

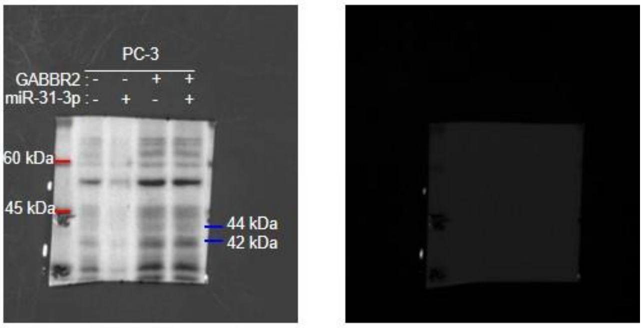

ERK

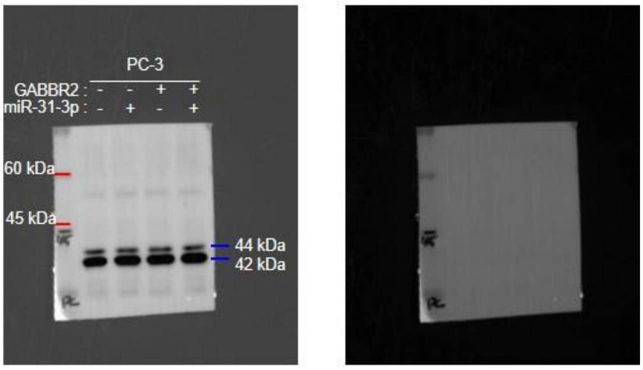

p-JNK

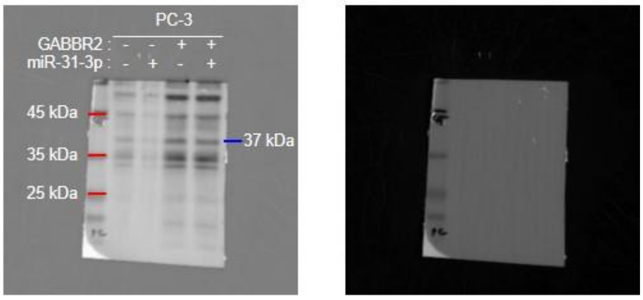

JNK

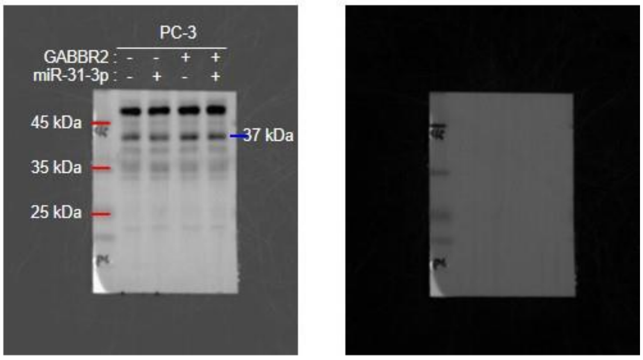

β-actin

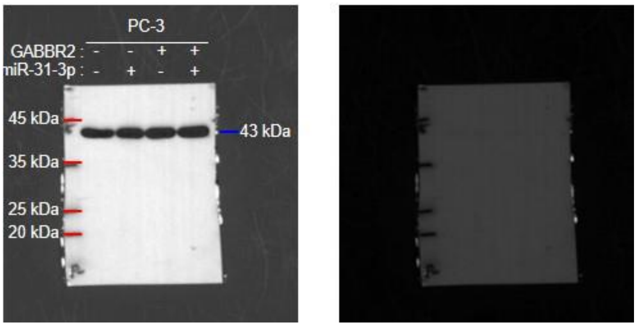

LNCap

GABBR2

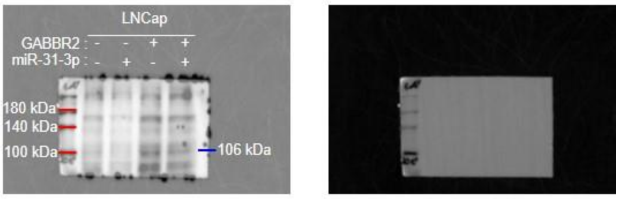

p-ERK

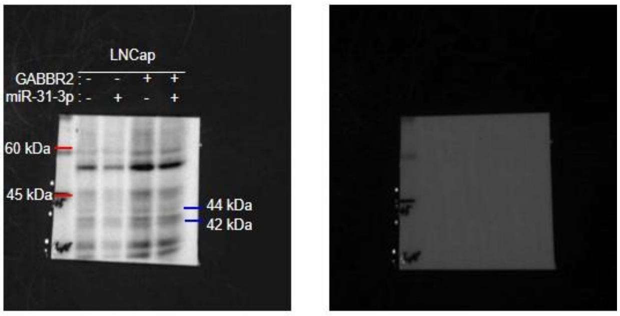

ERK

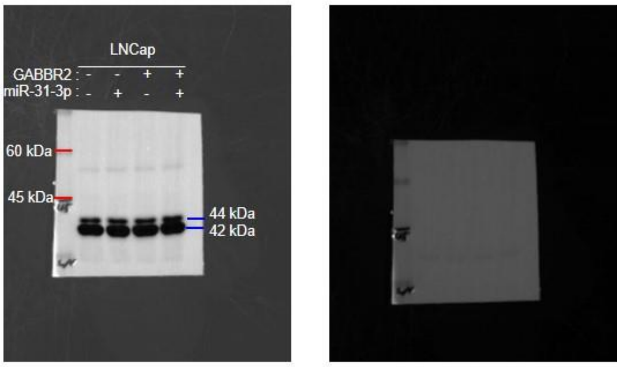

p-JNK

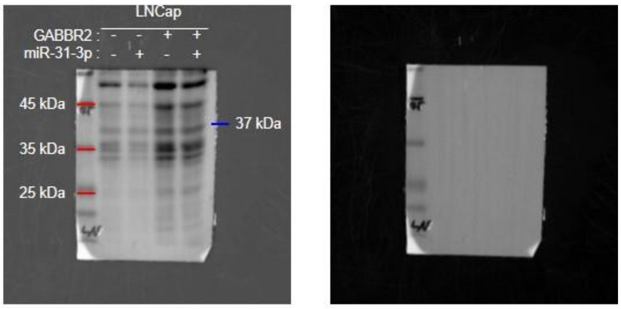

JNK

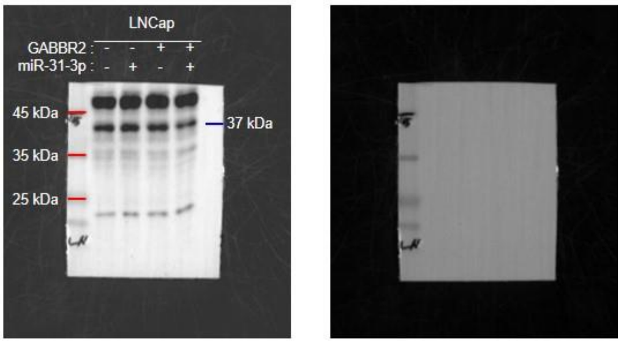

β-actin

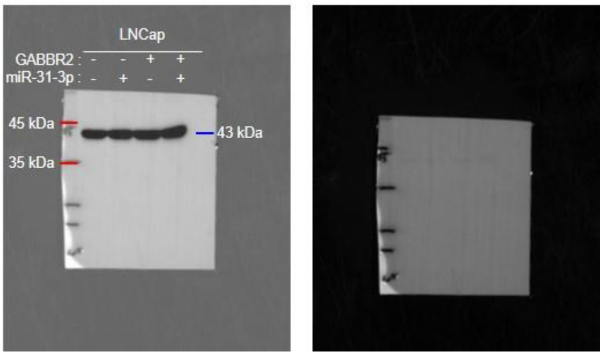

Supplement: Supplementary Figure 1 — Functional screening for miRNAs regulating PC cells growth. (A) miRNAs expression after transfection with indicated miRNA. Total RNA was prepared from transfected cells and indicated miRNA levels were analyzed by real-time polymerase chain reaction. RNU6B was used as the reference gene for normalization. An unpaired two-tailed Student’s t-test was used to calculate P values. Error bars represent mean ± standard error of the mean (SEM). ***P value < 0.001 vs. Cont (n = 3). (B) Representative image of morphology of PC cells transfected with the control miRNA, miR-4456, miR-646, miR-516a-5p, miR-941, miR-31-3p or miR-3181. (C) Identification of growth-inhibiting effects of miRNAs. Cell viability in cultured cells transfected with control miRNA, miR-4456, miR-646, miR-516a-5p, miR-941, miR-31-3p or miR-3181 was measured using Cell Counting Kit-8 at 72 h. Unpaired two-tailed Student’s t-test was used to calculate the P value. Error bars represent mean ± SEM. **P value < 0.01 and ***P value < 0.001 vs. Cont (n= 3). [file DataSheet_1.pdf]
